# Detection of the *PAX3-FKHR* fusion gene in paediatric rhabdomyosarcoma: a reproducible predictor of outcome?

**DOI:** 10.1054/bjoc.2001.2008

**Published:** 2001-09

**Authors:** J Anderson, T Gordon, A McManus, T Mapp, S Gould, A Kelsey, H McDowell, R Pinkerton, J Shipley, K Pritchard-Jones, (on behalf of the UK Children's Cancer Study Group (UKCCSG) and the UK Cancer Cytogenetics Group)

**Affiliations:** Section of Paediatric Oncology, Molecular Cytogenetics Team, Section of Molecular Carcinogenesis, Institute of Cancer Research, Sutton, Surrey, UK; United Kingdom Children's Cancer Study Group, Leicester, UK; Alder Hey Children's Hospital, Liverpool, UK; John Radcliffe Hospital, Oxford, UK; Manchester Children's Hospital, Manchester, UK

**Keywords:** rhabdomyosarcoma, prognosis, translocation

## Abstract

Rhabdomyosarcoma has 2 major histological subtypes, embryonal and alveolar. Alveolar histology is associated with the fusion genes *PAX3-FKHR* and *PAX7-FKHR*. Definition of alveolar has been complicated by changes in terminology and subjectivity. It is currently unclear whether adverse clinical behaviour is better predicted by the presence of these fusion genes or by alveolar histology. We have determined the presence of the *PAX3/7-FKHR* fusion genes in 91 primary rhabdomyosarcoma tumours using a combination of classical cytogenetics, FISH and RT-PCR, with a view to determining the clinical characteristics of tumours with and without the characteristic translocations. There were 37 patients with t(2;13)/PAX3-FKHR, 8 with t(1;13) PAX7-FKHR and 46 with neither translocation. One or other of the characteristic translocations was found in 31/38 (82%) of alveolar cases. Univariate survival analysis revealed the presence of the translocation t(2;13)/PAX3-FKHR to be an adverse prognostic factor. With the difficulties in morphological diagnosis of alveolar rhabdomyosarcoma on increasingly used small needle biopsy specimens, these data suggest that molecular analysis for PAX3-FKHR will be a clinically useful tool in treatment stratification in the future. This hypothesis requires testing in a prospective study. Variant t(1;13)/PAX7-FKHR appears biologically different, occurring in younger patients with more localised disease. © 2001 Cancer Research Campaignhttp://www.bjcancer.com


British Journal of Cancer (2001) **85**(6), 831–835
© 2001 Cancer Research Campaign
doi: 10.1054/ bjoc.2001.2008, available online at http://www.idealibrary.com on IDEAL

http://www.bjcancer.com

### Detection of the *PAX3-FKHR* fusion gene in paediatric rhabdomyosarcoma: a reproducible predictor of outcome?

**J Anderson[1*], T Gordon[1,2], A McManus[1,2], T Mapp[3], S Gould[5], A Kelsey[6], H McDowell[4], R Pinkerton[1], J Shipley[2] and K Pritchard-Jones[1] (on behalf of the UK Children's Cancer Study Group (UKCCSG) and the UK Cancer Cytogenetics Group)**

[1]Section of Paediatric Oncology and [2]Molecular Cytogenetics Team, Section of Molecular Carcinogenesis, Institute of Cancer Research, Sutton, Surrey, UK; [3]United Kingdom Children's Cancer Study Group, Leicester, UK; [4]Alder Hey Children's Hospital, Liverpool, UK; [5]John Radcliffe Hospital, Oxford, UK; [6]Manchester Children's Hospital, Manchester, UK

**Summary** Rhabdomyosarcoma has 2 major histological subtypes, embryonal and alveolar. Alveolar histology is associated with the fusion genes *PAX3-FKHR* and *PAX7-FKHR*. Definition of alveolar has been complicated by changes in terminology and subjectivity. It is currently unclear whether adverse clinical behaviour is better predicted by the presence of these fusion genes or by alveolar histology. We have determined the presence of the *PAX3/7-FKHR* fusion genes in 91 primary rhabdomyosarcoma tumours using a combination of classical cytogenetics, FISH and RT-PCR, with a view to determining the clinical characteristics of tumours with and without the characteristic translocations. There were 37 patients with t(2;13)/PAX3-FKHR, 8 with t(1;13) PAX7-FKHR and 46 with neither translocation. One or other of the characteristic translocations was found in 31/38 (82%) of alveolar cases. Univariate survival analysis revealed the presence of the translocation t(2;13)/PAX3-FKHR to be an adverse prognostic factor. With the difficulties in morphological diagnosis of alveolar rhabdomyosarcoma on increasingly used small needle biopsy specimens, these data suggest that molecular analysis for PAX3-FKHR will be a clinically useful tool in treatment stratification in the future. This hypothesis requires testing in a prospective study. Variant t(1;13)/PAX7-FKHR appears biologically different, occurring in younger patients with more localised disease. © 2001 Cancer Research Campaign http://www.bjcancer.com

**Keywords**: rhabdomyosarcoma; prognosis; translocation

Prognostic stratification in childhood cancer is the key to appropriate treatment. Clinical grouping is of continued value but the future lies in molecular characterisations that predict clinical behaviour. Use of multimodal therapy in childhood rhabdomyosarcoma, incorporating multiagent chemotherapy, has led to cure rates of over 60% (Crist et al, 1995). It has been established that older age at presentation, higher stage of disease and certain primary sites are adverse prognostic factors (Crist et al, 1990). Stratification of tumours into histological subgroups has been more controversial. For example, alveolar histology was identified as an independent adverse prognostic factor in the IRS (Intergroup Rhabdomyosarcoma Study) I and II studies (Crist et al, 1990), although this was not found in the IRS III study (Crist et al, 1995) probably because more aggressive treatment was given to alveolar tumours.

Identification of histological subtypes has been an evolving area that has led to the incorporation, into the alveolar category, of tumours with only minimal alveolar histology and/or the solid variant form of alveolar (Tsokos et al, 1992). Moreover a molecular correlate with the histological subdivision has been proposed whereby a characteristic reciprocal chromosomal translocation, t(2;13)(q35;q14), is considered diagnostic for alveolar histology (Scrable et al, 1989). The molecular changes involved in the t(2;13) translocation are well characterised and involve the disruption of the PAX3 transcription factor on chromosome 2 and its fusion in-frame with the transactivating 3′ end of the FKHR gene on chromosome 13 (Valentine et al, 1989; Galili et al, 1993; Bennicelli et al, 1995). A variant translocation, t(1;13)(p36;q14), is also found in alveolar rhabdomyosarcoma. The rearrangement is analogous and involves disruption of PAX7 on chromosome 1 and fusion to the FKHR transcription activation domain (Davis et al, 1994). There is emerging evidence that the 2 rearrangements are associated with distinct clinical phenotypes; the t(2;13)(q35;q14) cases occur in older patients and have a more aggressive behaviour (Kelly et al, 1997).

The cloning of the genes at the breakpoints of these translocations has allowed the development of molecular techniques to supplement classical cytogenetics in the diagnosis and classification of small round cell tumours. Both fluorescence in situ hybridisation (FISH) (McManus et al, 1996b) and reverse transcriptase–polymerase chain reaction (RT-PCR) (Downing et al, 1995; Anderson et al, 1997) can be used on snap-frozen tumours to indicate disruption of the genes and expression of the fusion transcripts. The discovery of characteristic translocations associated with other small round cell tumours means that these techniques

*Received 20 November 2000*
*Revised 29 May 2001*
*Accepted 3 July 2001*

*Correspondence to:* K Pritchard-Jones

*Current address: Unit of Molecular Haematology and Oncology, Institute of Child Health, Guilford Street, London WC1N 1EH, UK.

also have a role to play in differential diagnosis and the development of new molecular classifications.

Previous studies (Douglass et al, 1993) to define the role of reciprocal translocations in rhabdomyosarcoma have been limited by difficulties in establishing karyotypes and by the limited availability of frozen tissue for FISH or RT-PCR. We have developed an RT-PCR assay, which works on formalin-fixed tissue (Anderson et al, 1997) and allows a more extensive study of archival material from patients for whom there are detailed data of treatment and outcome. We have analysed a group of 91 rhabdomyosarcomas for the presence of the *PAX3-FKHR* and the *PAX7-FKHR* fusions using a combination of the techniques of RT-PCR, FISH and classical cytogenetics, and correlated these results with tumour phenotype and clinical outcome. Because of the changes in definition of alveolar histology over time, it was problematic to correlate histological type, molecular findings and clinical outcome. Re-review of histology of the entire tumour set has proved difficult due to sample availability. This study therefore presents only a correlation between molecular pathology and outcome, rather than a comparison of genotype with histology. Molecular pathology has the advantage of being less subjective, particularly with small samples.

### PATIENTS AND METHODS

### Patient population and eligibility

Survival analysis was performed on 91 patients who had unequivocal evidence for the presence or absence of the characteristic fusion genes. Patient selection was therefore determined by the availability of material for molecular analysis and/or whether the tumour had produced successful metaphase chromosomes. All were treated in UK children's cancer centres between 1982 and 1997, and had a clear histological diagnosis of rhabdomyosarcoma. Analysed patients were aged between 4 months and 23 years with a mean of 7 and a median of 5 years. Three patients were aged over 18 years but were treated on paediatric chemotherapy regimens. Of analysed patients, 63 patients were boys and 28 were girls. 45 (49%) were treated with the SIOP (International Society of Paediatric Oncology) MMT89 (malignant mesenchymal tumour) protocol and 22 (24%) with the closely related MMT95 protocol, representing 19% of all UK patients entered in these 2 trials. 5 patients were treated before 1989 in the Royal Marsden Hospital using the Rapid VAC protocol (Pinkerton et al, 1991) and 19 others (20%) were treated since 1989 in UK paediatric cancer centres using other multimodality and multiagent chemotherapy protocols of similar intensity to the MMT regimens. All patients were treated with a combination of drugs including vincristine, ifosfamide (or cyclophosphamide), and actinomycin D. Higher stage tumours and some alveolar tumours additionally received epirubicin, etoposide and carboplatin. All patients were treated with intensive chemotherapy and surgery, with or without radiotherapy for local control. High-dose therapy with bone marrow or stem cell rescue in first remission was limited to metastatic patients or patients with stage 3 disease and alveolar histology.

Survival and outcome data were obtained from the UKCCSG data centre, Leicester, UK for the 74% of patients treated on the SIOP MMT protocols. Records of non-MMT patients were retrieved from participating hospitals' own databases.

### Molecular and cytogenetic analysis

Cytogenetic data were available from 46 rhabdomyosarcoma cases as a separate study surveying the karyotypes of this tumour group on behalf of the United Kingdom Cancer Cytogenetics Group (Gordon et al, 2001) including data from 7 previously published cases (Roberts et al, 1992; Bown et al, 1994; Yule et al, 1995; McManus et al, 1996a; Weber-Hall et al, 1996). 35 of these cases were included on the basis of unequivocal evidence for the presence or absence of the rearrangements associated with the known fusion genes and breakpoints at the *PAX3* or *PAX7* and *FKHR* loci. Cytogenetic evidence for absence of the fusion genes required complete abnormal karyotypes with no rearrangements or double minutes, which can be associated with fusion gene amplification or cryptic translocations. 3 further cases with double minutes were subsequently included because molecular analysis indicated the presence of the *PAX7-FKHR* fusion. 11 cases were included on the basis of unequivocal cytogenetic data alone. Suitable material was available for analysis by another methodology in 16/20 cases with and 11/18 cases without a translocation and there was complete concordance between the cytogenetic and molecular results.

80 tumours had material available for molecular studies, either as freshly resected samples (for making touch imprints and then freezing), as snap-frozen resections or biopsies, or as formalin-fixed, paraffin-embedded tissue. The method of analysis was RT-PCR on RNA extracted from frozen ($n$ = 47) or fixed ($n$ = 39) material or interphase FISH ($n$ = 29). In only 35 tumours did the translocation status rely on a single molecular test (RT-PCR on frozen ($n$ = 16) or fixed ($n$ = 19) tissue). 29 tumours were analysed by 2 methods and 16 by 3 methods, again with complete concordance of translocation status.

Interphase and metaphase FISH studies for the rhabdomyosarcoma translocations were performed using chromosome 13 cosmids flanking the *FKHR* gene as previously described (Biegel et al, 1995). Cosmids were labelled with rhodamine-4-dUTP or fluorescein-11-dUTP, respectively, so that intact chromosomes 13 in interphase and metaphase nuclei would be seen as red/green doublets whilst disruption would result in separation of the signals. A minimum of 50 images were captured and examined per tumour and interpretation of results was performed with observer blinding to clinical details of the cases. All FISH results were confirmed by another method. The identity of the partner for *FKHR* in cases with disruption was determined by RT-PCR.

Fixed tissue which was used for RT-PCR was cut from blocks which contained predominantly tumour. Likewise, the pathology reports of the blocks taken adjacent to the snap-frozen samples were reviewed to confirm that the latter were predominantly tumour. RNA was prepared using Trizol reagent (GIBCO BRL, Gaithersburg MD) as previously described (Anderson et al, 1997). 1 μg of RNA was reverse transcribed using random hexamer priming and Superscript II reverse transcriptase (GIBCO BRL) according to the manufacturer's instructions. The resulting cDNAs from fixed tumour were amplified for *PAX3-FKHR* and *PAX7-FKHR* by nested RT-PCR using previously published primers and conditions (Anderson et al, 1997). RNA from snap-frozen tumour was assayed by single round RT-PCR using the following novel primer pairs and conditions: (PAX3-FKHR forward gcactgtacaccaaagcacg, reverse ctgtggattgagcatccacc, annealing 60˚C and 1.5 mM $MgCl_2$; PAX7-FKHR forward aaccacatgaacccggtcag, reverse aactgtgatccagggctgtc, annealing 55˚C, and 1.5 mM $MgCl_2$, 30 cycles). The expression of the housekeeper genes β-actin and/or

 *© 2001 Cancer Research Campaign*

18S RNA was used as a positive control for the presence of amplifiable RNA. Amplified RT-PCR products were separated on 2% ethidium-stained agarose gels and the identity of the fusion gene confirmed by automated sequencing and/or hybridisation to internal PAX3-or PAX7-specific oligonucleotide probes. 'No RNA' and 'no cDNA' negative controls were included in each assay. The methodology for formalin-fixed tissue using nested PCR has been described previously (Anderson et al, 1997). Briefly, formalin-fixed tumour samples known to contain either the *PAX3-FKHR*, *PAX7-FKHR* or no fusion gene (based on analysis of frozen aliquots of the same tumour) were used as controls. cDNA synthesised from test formalin-fixed tumour samples and controls were included in each assay and used as templates for amplification of both the FKHR fusions and 2 housekeeper genes (actin and/or 18S RNA) in a nested PCR reaction. The quality of RNA extracted from each fixed tumour sample was deemed to be informative for analysis of its *PAX3/7-FKHR* status by RT-PCR if the housekeeper gene product measured after 22 cycles was of equivalent or greater intensity to the controls (a cycle number of 22 for amplification of the housekeeper genes had been shown previously to result in the best discrimination of RNA quality). PCR product separation and identification were performed as for frozen samples. Quantitation of PCR products was performed by measuring band intensity on an ethidium bromide-stained agarose gel using NIH software. For all pre-PCR stages, but especially those involving formalin-fixed tissue, strict precautions were taken to prevent DNA contamination of reactions.

Just under 50% of fixed tumour blocks analysed by RT-PCR (39/79) gave informative results. 20 of these had at least one other additional technique performed, all showing concordance of results. The clinical characteristics of the 40 cases from which RNA could not be amplified were similar to those with good quality RNA, hence this criterion should not have introduced any bias.

### Statistical analysis

Survival data were collected after the completion of the molecular analysis. Event-free survival was defined as the time from diagnosis until the first occurrence of an event (relapse, death or disease progression) or last clinical contact; and overall survival was estimated in terms of time from diagnosis until death or the date of last clinical contact. Overall survival and event-free survival were estimated by the method of Kaplan and Meier.

### RESULTS

### Occurrence of the t(2; 13) & t(1; 13) translocations according to clinical phenotype

There were 37 tumours with t(2;13)/PAX3-FKHR, 8 with t(1;13)/PAX7-FKHR and 46 with neither (Table 1). Overall, more than one methodology was used in 45 cases. There was concordance of results in these cases justifying the inclusion of patients where only one methodology was used. Sequencing of PCR products from the t(1;13)/PAX7-FKHR and t(2;13)/PAX3-FKHR assays showed no variability in breakpoints or sequence.

There is a statistically significant association between t(2;13)/PAX3-FKHR and higher-stage disease and between the absence of a translocation and lower-stage disease ($P$ = 0.006, Fischer's exact test, when grouping SIOP stages 1 & 2 and stages 3 & 4). The numbers of cases with the t(1;13)/PAX7-FKHR translocation is too small to reach statistical significance although an observable trend suggests that they are more akin to the cases with neither translocation in terms of clinical stage (Table 1). Similarly, the median ages are significantly different with the t(2;13)/PAX3-FKHR being seen in older children than either the t(1;13)/PAX7-FKHR or neither translocation. These median ages are 9, 3 and 4 years respectively and the age differences are significant ($P$ = 0.001 Kruskal–Wallis Test). There is no significant association between translocation status and disease site.

**Table 1** Clinical features of patients with characteristic translocations and patients lacking these translocations.

| | Number | Histology* | Gender m/f | Median age | Sites | Favourable/unfavourable sites | SIOP stage | % stages 1&2 | % stages 3&4 |
|---|---|---|---|---|---|---|---|---|---|
| t(1;13) | 8 | alv 6<br>emb 1<br>rms-nos 1 | 5/3 | 3 | gen 1<br>h&n 3<br>orb 1<br>limb 3 | 5/8=64% | 1=3<br>2=2<br>3=1<br>4=2 | 62 | 38 |
| t(2;13) | 37 | alv 25<br>emb 8<br>rms-nos 3<br>rms-undiff 1 | 27/10 | 9 | gen 2<br>h&n 4<br>orbit 2<br>hep 4<br>limb 12<br>perineum 2<br>p/m 2<br>syst 2<br>trunk 7 | 8/37=22% | 1=10<br>2=4<br>3=8<br>4=15 | 37 | 63 |
| Neither | 46 | alv 7<br>emb 34<br>rms-nos 5 | 31/15 | 4 | gen 9<br>h&n 5<br>limb 10<br>p/m 9<br>syst 1<br>thor 1<br>trunk 3<br>blad 5 | 14/46=30% | 1=20<br>2=13<br>3=5<br>4=8 | 72 | 28 |

Favourable sites are defined as genitourinary (non bladder prostate) and head and neck (non parameningeal). Gen = genitourinary; h&n = head and neck; p/m = parameningeal; syst = systemic; thor = thoracic; blad = bladder; hep = hepatobiliary; orb = orbital. *Histological subtype is that of the original central pathology review (when carried out) or the local pathologist's report: alv = alveolar; emb = embryonal; nos = not otherwise specified; undiff = undifferentiated.

© 2001 Cancer Research Campaign 

A

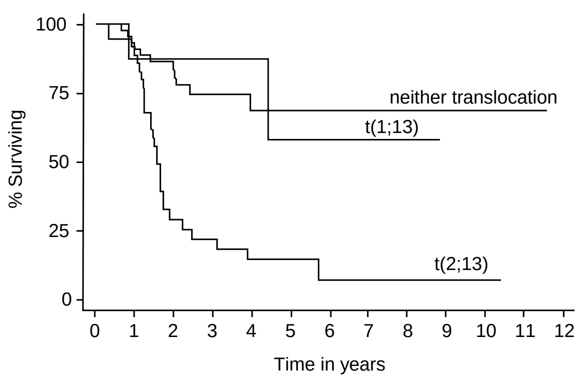

**Figure 1A** Overall survival by translocation status. Significantly poorer survival ($P < 0.0001$) for t(2;13) as compared to neither translocation

B

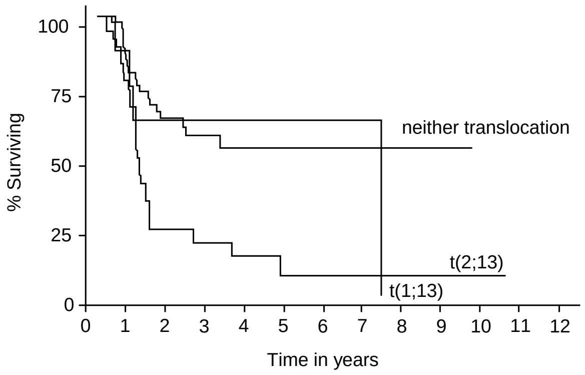

**Figure 1B** Event-free survival by translocation status. Significantly poorer survival ($P < 0.0001$) for t(2;13) as compared to neither translocation

C

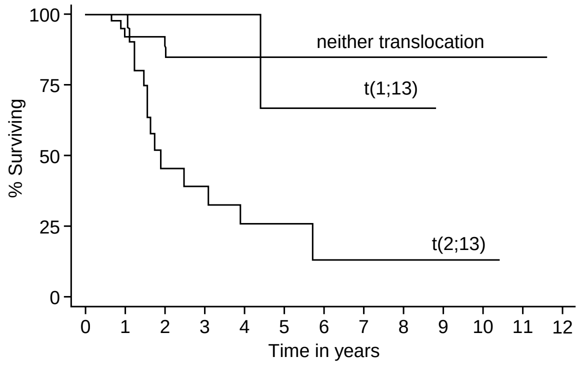

**Figure 1C** 66 stage 1–3 patients. Overall survival by translocation status. Significantly poorer survival ($P < 0.0001$) for t(2;13) as compared to neither translocation

D

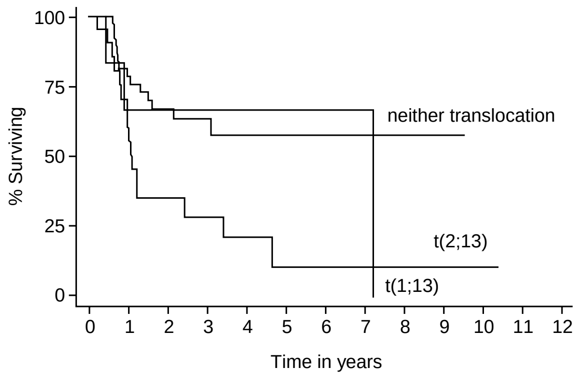

**Figure 1D** 66 stage 1–3 patients. Event-free survival by translocation status. Significantly poorer survival ($P = 0.005$) for t(2;13) as compared to neither translocation

### Survival analysis

The median follow-up for all 91 cases is 3.4 years. In univariate analysis of all patients, age, stage, histological risk group and presence of t(2;13)/PAX3-FKHR translocation were statistically significant adverse prognostic factors for event-free and overall survival (Figure 1 A, B and data not shown). We were interested in the prognostic impact of translocations amongst patients with localised disease where treatment intensification is an option. Among 66 non-metastatic patients, overall and event-free survival were significantly worse in the patients with t(2;13)/PAX3-FKHR than in those without (Figure 1C, D).

### DISCUSSION

This study describes the largest analysis to date of the clinical impact of characteristic chromosomal translocations in childhood rhabdomyosarcoma. Survival analysis indicates that the presence of a t(2;13)/PAX3-FKHR is an adverse prognostic factor. The majority (74%) of patients were treated using the same SIOP MMT protocols, and non-MMT patients were treated in a broadly similar manner. More aggressive first-line therapy was given to some of the more recently diagnosed patients with alveolar histology, most of whom had t(2;13)/PAX3-FKHR. If anything, this may have led to an underestimate of the prognostic impact of the t(2;13)/PAX3-FKHR translocation. The comparative prognostic value of this tumour-specific translocation compared with alveolar histology needs to be assessed in a prospective study with uniform pathological diagnostic criteria and central review.

The number of cases with the variant translocation t(1;13)/PAX7-FKHR was too small to allow statistically significant analyses. However, the picture which is emerging from this and other studies (Kelly et al, 1997) is that these tumours have classical alveolar histology but a different clinical phenotype including a younger age at presentation, lower disease staging and better prognosis. Thus they appear biologically distinct from the t(2;13)/PAX3-FKHR tumours despite indistinguishable histological features.

There is a disproportionately high incidence of alveolar histology in the study for 2 reasons. First, because of the inclusion of tumours with karyotype information and therefore the automatic exclusion of cases in which cytogenetic analysis was unsuccessful, not attempted or equivocal. Second, because of the high incidence of extremity primaries, which are more likely to be of the alveolar subtype and which are more likely to have either primary surgery or a large biopsy, providing sufficient material for molecular analysis. This selection is likely to occur to a similar extent in prospective studies, largely because of the trend towards minimally invasive diagnostic procedures and the deferment of definitive surgery.

© 2001 Cancer Research Campaign

With the increasing use of minimal biopsies and a move away from up-front surgery, it seems likely that small areas of alveolar architecture in an otherwise typical embryonal or undifferentiated tumour might be missed despite rigorous histological examination of the biopsy specimens. The identification of the presence of the t(2;13) translocation/PAX3-FKHR fusion as an adverse prognostic factor may have an impact on treatment in such cases. Prospective studies are needed, incorporating rigorous application of uniform diagnostic histological criteria and molecular analyses, to confirm our findings and clarify the relative prognostic significance of t(1;13)/PAX7-FKHR, t(2;13)/PAX3-FKHR and alveolar histology. Ultimately an understanding of how the fusion proteins contribute to tumorigenesis and the aggessive behaviour of the alveolar subtype may suggest novel treatment approaches.

### ACKNOWLEDGEMENTS

We thank all of the clinicians and pathologists who provided tumour material for this study, in particular Dr G Kokai, Alder Hey Children's Hospital, Liverpool; Prof A Malcolm and Dr J Hale, Royal Victoria Infirmary, Newcastle upon Tyne; Dr C Cullinane and Dr M Michelagnoli, St James University Hospital, Leeds; Dr F Raafat, Birmingham Children's Hospital; Dr C Mitchell, John Radcliffe Infirmary, Oxford; Dr I Moore, Southampton General Hospital; Dr V Smith, Dublin; Dr A Howatson, Royal Hospital for Sick Children, Glasgow; Dr A Charles and Dr S Lowis, Bristol Royal Hospital for Sick Children; Dr I Jeffrey, St George's Hospital, London; Prof C Cooper, The Institute of Cancer Research, Sutton; Dr C Fisher, The Royal Marsden Hospital, London. We thank Professor Richard Carter for his expert contribution to the pathology review. This work was supported by the Cancer Research Campaign, the Medical Research Council of the United Kingdom and the Royal Marsden Hospital Children's Cancer Unit Fund.

### REFERENCES

Anderson J, Renshaw J, McManus A, Carter R, Mitchell C, Adams S and Pritchard-Jones K (1997) Amplification of the t(2; 13) and t(1; 13) translocations of alveolar rhabdomyosarcoma in small formalin-fixed biopsies using a modified reverse transcriptase polymerase chain reaction. *Am J Pathol* **150**: 477–482

Bennicelli JL, Fredericks WJ, Wilson RB, Rauscher FJ, 3rd and Barr FG (1995) Wild type PAX3 protein and the PAX3-FKHR fusion protein of alveolar rhabdomyosarcoma contain potent, structurally distinct transcriptional activation domains. *Oncogene* **11**: 119–130

Biegel JA, Nycum LM, Valentine V, Barr FG and Shapiro DN (1995) Detection of the t(2;13)(q35;q14) and PAX3-FKHR fusion in alveolar rhabdomyosarcoma by fluorescence in situ hybridization. *Genes Chromosomes Cancer* **12**: 186–192

Bown NP, Reid MM, Malcolm AJ, Davison EV, Craft AW and Pearson AD (1994) Cytogenetic abnormalities of small round cell tumours. *Med Pediatr Oncol* **23**: 124–129

Crist W, Gehan EA, Ragab AH, Dickman PS, Donaldson SS, Fryer C, Hammond D, Hays DM, Herrmann J, Heyn R et al (1995) The Third Intergroup Rhabdomyosarcoma Study. *J Clin Oncol* **13**: 610–630

Crist WM, Garnsey L, Beltangady MS, Gehan E, Ruymann F, Webber B, Hays DM, Wharam M and Maurer HM (1990) Prognosis in children with rhabdomyosarcoma: a report of the intergroup rhabdomyosarcoma studies I and II. Intergroup Rhabdomyosarcoma Committee [see comments]. *J Clin Oncol* **8**: 443–452

Davis RJ, D'Cruz CM, Lovell MA, Biegel JA and Barr FG (1994) Fusion of PAX7 to FKHR by the variant t(1;13)(p36;q14) translocation in alveolar rhabdomyosarcoma. *Cancer Res* **54**: 2869–2872

Douglass EC, Shapiro DN, Valentine M, Rowe ST, Carroll AJ, Raney RB, Ragab AH, Abella SM and Parham DM (1993) Alveolar rhabdomyosarcoma with the t(2;13): cytogenetic findings and clinicopathologic correlations. *Med Pediatr Oncol* **21**: 83–87

Downing JR, Khandekar A, Shurtleff SA, Head DR, Parham DM, Webber BL, Pappo AS, Hulshof MG, Conn WP and Shapiro DN (1995) Multiplex RT-PCR assay for the differential diagnosis of alveolar rhabdomyosarcoma and Ewing's sarcoma. *Am J Pathol* **146**: 626–634

Galili N, Davis RJ, Fredericks WJ, Mukhopadhyay S, Rauscher FJD, Emanuel BS, Rovera G and Barr FG (1993) Fusion of a fork head domain gene to PAX3 in the solid tumour alveolar rhabdomyosarcoma [published erratum appears in Nat Genet 1994 Feb; 6(2): 214]. *Nat Genet* **5**: 230–235

Gordon AT, McManus A, Anderson J, Min T, Swansbury J, Pritchard-Jones K and Shipley J (2001) Cytogenetic Abnormalities in 42 Rhabdomyosarcoma. A United Kingdom Cancer Cytogenetics Group Study. *Med Ped Oncol* **36**: 259–267

Kelly KM, Womer RB, Sorensen PH, Xiong QB and Barr FG (1997) Common and variant gene fusions predict distinct clinical phenotypes in rhabdomyosarcoma. *J Clin Oncol* **15**: 1831–1836

McManus AP, Min T, Swansbury GJ, Gusterson BA, Pinkerton CR and Shipley JM (1996a) der(16)t(1;16)(q21;q13) as a secondary change in alveolar rhabdomyosarcoma. A case report and review of the literature. *Cancer Genet Cytogenet* **87**: 179–181

McManus AP, O'Reilly MA, Jones KP, Gusterson BA, Mitchell CD, Pinkerton CR and Shipley JM (1996b). Interphase fluorescence in situ hybridization detection of t(2;13)(q35;q14) in alveolar rhabdomyosarcoma–a diagnostic tool in minimally invasive biopsies. *J Pathol* **178**: 410–414

Pinkerton CR, Groot-Loonen J, Barrett A, Meller ST, Tait D, Ashley S and McElwain TJ (1991) Rapid VAC high dose melphalan regimen, a novel chemotherapy approach in childhood soft tissue sarcomas. *Br J Cancer* **64**: 381–385

Roberts P, Browne CF, Lewis IJ, Bailey CC, Spicer RD, Williams J and Batcup G (1992) 12q13 abnormality in rhabdomyosarcoma. A nonrandom occurrence? *Cancer Genet Cytogenet* **60**: 135–140

Scrable H, Witte D, Shimada H, Seemayer T, Sheng WW, Soukup S, Koufos A, Houghton P, Lampkin B and Cavenee W (1989) Molecular differential pathology of rhabdomyosarcoma. *Genes Chromosomes Cancer* **1**: 23–35

Tsokos M, Webber BL, Parham DM, Wesley RA, Miser A, Miser JS, Etcubanas E, Kinsella T, Grayson J, Glatstein E et al (1992) Rhabdomyosarcoma. A new classification scheme related to prognosis. *Arch Pathol Lab Med* **116**: 847–855

Valentine M, Douglass EC and Look AT (1989) Closely linked loci on the long arm of chromosome 13 flank a specific 2;13 translocation breakpoint in childhood rhabdomyosarcoma. *Cytogenet Cell Genet* **52**: 128–132

Weber-Hall S, McManus A, Anderson J, Nojima T, Abe S, Pritchard-Jones K and Shipley J (1996) Novel formation and amplification of the PAX7-FKHR fusion gene in a case of alveolar rhabdomyosarcoma. *Genes Chromosomes Cancer* **17**: 7–13

Yule SM, Bown N, Malcolm AJ, Reid MM and Pearson AD (1995) Solid alveolar rhabdomyosarcoma with a t(2;13). *Cancer Genet Cytogenet* **80**: 107–109

*© 2001 Cancer Research Campaign* 
